# Effect of Perioperative Lidocaine, Propofol and Steroids on Pulmonary Metastasis in a Murine Model of Breast Cancer Surgery

**DOI:** 10.3390/cancers11050613

**Published:** 2019-05-01

**Authors:** James Freeman, Peter D. Crowley, Andrew G. Foley, Helen C. Gallagher, Masae Iwasaki, Daqing Ma, Donal J. Buggy

**Affiliations:** 1 Department of Anaesthesia, Mater University Hospital, Dublin 7, Ireland; donal.buggy@ucd.ie; 2 Conway Institute for Biomedical Sciences, School of Medicine, University College Dublin, Dublin 4, Ireland; peter.crowley@ucdconnect.ie (P.D.C.); helen.gallagher@ucd.ie (H.C.G.); 3 Berand Neuropharmacology, NovaUCD, Belfield Innovation Park, University College Dublin, Dublin 4, Ireland; andrew.foley@berand.ie; 4 Anaesthetics, Pain Medicine and Intensive Care, Department of Surgery and Cancer, Faculty of Medicine, Imperial College London, Chelsea and Westminster Hospital, London SW10 9NH, UK; masae-a@nms.ac.jp (M.I.); d.ma@imperial.ac.uk (D.M.); 5 Outcomes Research, Cleveland Clinic, Cleveland, OH 44195, USA

**Keywords:** cancer recurrence, cancer metastasis, anaesthesia inhalation, anaesthesia intravenous propofol, steroid methylprednisolone, local anaesthetics, lidocaine

## Abstract

Addressing the hypothesis that anaesthetic-analgesic technique during cancer surgery might influence recurrence or metastatic spread is a research priority. Propofol, which has anti-inflammatory properties in vitro, is clinically associated with reduced risk of cancer recurrence compared with sevoflurane anaesthesia in retrospective studies. Amide local anaesthetics, such as lidocaine, have cancer inhibiting effects in vitro. Steroids have anti-inflammatory and immunosuppressive effects and are associated with improved recovery after major non-cancer surgery. We compared the effects of propofol, lidocaine and methylprednisolone on postoperative metastasis in a murine model of breast cancer surgery under sevoflurane anaesthesia. 4T1 tumour cells were introduced into the mammary fat-pad of female BALB/c mice and the resulting tumour resected seven days later under general anaesthesia with sevoflurane. Mice (*n* = 72) were randomized to four treatment groups: Sevoflurane alone (control); Propofol group received 5 mg.kg^−1^; Lidocaine group received 1.5 mg.kg^−1^ followed by 2 mg.kg^−1^.h^−1^ infusion; Methylprednisolone group received 30 mg.kg^−1^ methylprednisolone. The primary outcome measure was pulmonary metastasis colony count, as assessed by in-vitro proliferation, two weeks post-operatively. This was achieved by treating the post-mortem lung tissue with collagenase IV, straining and culturing for 14 days prior to colony count. Compared with control, lidocaine and propofol each individually reduced pulmonary metastasis colonies; mean (SD) 846 (±581) vs. 88 (±52) vs. 34 (±44) respectively, (*p* = 0.0001 and *p* = 0.0001). Methylprednisolone increased lung metastasis, 2555 (±609) vs. 846 (±581), *p* = 0.0001. Post-operative hepatic metastatic disease and serum interleukin-6 and vascular endothelial growth factor levels were similar in all groups. In conclusion, in a murine model of breast cancer surgery during sevoflurane anaesthesia, propofol and lidocaine each decreased pulmonary metastasis, while methylprednisolone increased it.

## 1. Introduction

Malignant breast disease remains an important cause of cancer death in women [1]. Metastatic disease accounts for the majority of breast cancer-related deaths [2] and the implementation of focused efforts aimed at reducing the burden of metastatic breast disease would benefit not just the affected individual, but also provide an economic dividend for society. Despite the development of adjuvant therapies [3], surgery is offered to over 80% of breast cancer patients [4] and remains the principal element around which treatment strategies are developed.

It is more than a decade since initial retrospective clinical analyses suggested that perioperative interventions during cancer surgery, including anaesthetic technique, might modify post-operative tumour recurrence and metastasis risk [5,6]. In vitro analysis indicates a variety of mechanisms by which perioperative interventions could impact recurrence or metastasis [7,8,9]. A 2015 British Journal of Anaesthesia workgroup highlighted the paucity of in vivo evidence for this hypothesis [10]. Using a murine model of breast cancer, we have previously demonstrated that perioperative intravenous lidocaine reduces the pulmonary metastatic tumour load after primary tumour resection under inhalational anaesthesia with sevoflurane [11].

Propofol is the most widely used intravenous anaesthetic agent in modern anaesthetic practice and there is in vitro and retrospective clinical evidence that propofol has potentially protective effects against cancer cell invasion [12,13,14]. The possible underpinning mechanisms may be immunological [8,14,15,16], anti-inflammatory [17,18] or anti-angiogenic [19], all of which are distinct from the central effects of propofol administration. In a recent retrospective analysis of >5000 patients, propofol total intravenous anaesthesia was associated with a reduced risk of cancer recurrence when compared to inhalational anaesthesia [13].

Steroids are also anti-inflammatory but immunosuppressive [20]. Single perioperative doses have improved some postoperative outcomes after major non-cancer surgeries [21,22,23] but there is scant in vivo evidence as to whether the balance between its anti-inflammatory and immunosuppressive effects is beneficial or detrimental in post tumour surgery metastasis. 

Accordingly, we tested the hypotheses that, in a murine model of breast cancer, intravenous propofol and methylprednisolone reduce post-operative metastasis to a similar extent as lidocaine during sevoflurane anaesthesia due to anti-inflammatory and anti-angiogenic effects. 

## 2. Results

Unless otherwise stated, results are given as mean and standard deviation (SD). A probability value of less than 0.05 was considered statistically significant. 

### 2.1. Baseline Characteristics

Throughout the four groups, 72 animals were inoculated. Six animals did not develop observable tumours, three were found to have extensive intraperitoneal disease at the time of primary excision and one animal scored excessively high on welfare checks post-operatively. These ten animals were humanely euthanised and were not included in any analysis. Data for two animals were unavailable due to inadvertent damage to post-mortem specimen plates. As a result, 60 animals were available for inclusion in analysis, with 15 animals in each experimental group. See Table 1. 

**Figure 1:**
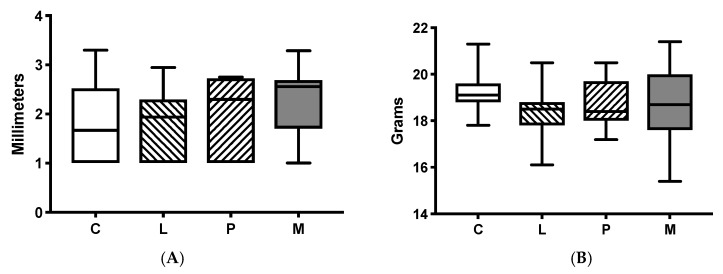
Mean tumour diameters and weights of each intervention group. (**A**) Tumour diameter, in millimetres, at time of surgical resection on study day 7. There were no significant differences between the experimental groups. Median, interquartile range and full range is displayed. The tumour diameter was calculated by using the square root of the values obtained when the tumour was measured in two different axes. C= control group. L = lidocaine group. P = propofol group. M = methylprednisolone group. (**B**) Mouse weight, in grams, prior to tumour resection on study day 7. Median, interquartile range and full range is displayed There were no significant differences between the experimental groups. C = control group. L = lidocaine group. P = propofol group. M = methylprednisolone group.

The mean tumour diameters and weights of each intervention group included in the final statistical analysis can be found in Figure 1. There were no significant differences in weight (*p* = 0.24) or tumour diameter (*p* = 0.23) between the treatment groups at the time of tumour resection. The mean (SD) tumour diameters in the Control group, Lidocaine group, Propofol group, and Methylprednisolone group were 1.84 cm (0.84), 1.78 cm (0.68), 2.04 cm (0.7), and 2.29 cm (0.74) respectively. The respective group mean (SD) weighs were 19.24 g (0.93), 18.41 g (1.18), 18.79 g (1.02), and 18.77 g (1.49). 

### 2.2. Pulmonary Colony Counts

**Figure 2:**
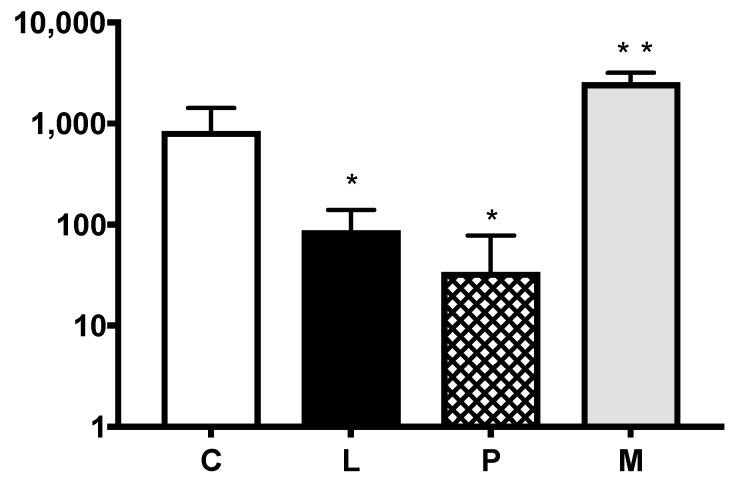
Pulmonary metastatic colonies. The x-axis represents the metastatic colonies counted in lung tissue samples. Respectively, Control group, Lidocaine group, Propofol group and Methylprednisolone group had means (SD) of 846 (±581), 88 (±52), 34 (±44) and 2555 (±609) pulmonary colonies counted. There was a significant reduction in pulmonary metastatic colonies between the Control group and the Lidocaine group, *p* = 0.0001 and the Propofol group, *p* = 0.0001. We found a significantly higher metastatic burden to the lungs in the Methylprednisolone group when compared to control, *p* = 0.0001. Colony counts are represented by a logarithmic scale in order to better display data. C= control group. L = lidocaine group. P = propofol group. M = methylprednisolone group. * *p* = significantly reduced colony count when compared to control group. ** *p* = significantly increased colony count when compared to control group.

The pulmonary colony counts in each of the groups can be found in Figure 2. Respectively, Control group, Lidocaine group, Propofol group, and Methylprednisolone group had means (SD) of 846 (±581), 88 (±52), 34 (±44), and 2555 (±609) pulmonary colonies counted. Intravenous lidocaine significantly reduced the pulmonary metastatic burden when compared to the control, with mean (SD) colony counts of 87 (±63) and 846 (±581) respectively, *p* = 0.0001. Intravenous propofol administration had a similarly protective effect with a reduction in colonies to a mean (SD) of 34 (±44) from the control of 846 (±581), *p* = 0.0001. Perioperative administration of high dose methylprednisolone had a detrimental effect on pulmonary metastatic disease with a significant increase in mean metastatic colonies when compared to control, 2555 (±609) vs. 846 (±581), *p* = 0.0001. 

### 2.3. Hepatic Colony Counts

Respectively, the Control group, Lidocaine group, Propofol group, and Methylprednisolone group had means (SD) of 74.7 (±257.3), 77.3 (±189.5), 55.3 (±189.6) and 176 (±375.1) liver metastatic colonies counted. There were no significant differences between any of the groups. 

### 2.4. Cytokine Analysis

No statistical differences in serum interleukin-6 (IL-6) or vascular endothelial growth factor (VEGF) concentrations were found between any of the experimental groups after a one-way ANOVA was employed with analysis of variance and post hoc Bonferroni correction for multiple testing. Mean (SD) serum VEGF concentrations, in pg.mL^−1^, across the different experimental groups were as follows: Control group 2068 (±3007), Lidocaine group 227.3 (±120.3), Propofol group 286.5 (±90.53) and Methylprednisolone group 383.1 (±237). Mean (SD) serum IL-6 values, in pg.mL^−1^, were 17.5 (±20.1), 8.21 (±14.22), 60.9 (±34.3) and 70.2 (±39.6) in the Control group, Lidocaine group, Propofol group, and Methylprednisolone group respectively. 

## 3. Discussion

In a murine model of breast cancer surgery, undertaken during sevoflurane anaesthesia, the perioperative administration of lidocaine, propofol and methylprednisolone influences pulmonary metastatic burden. There were no differences between the experimental groups with respect to hepatic metastatic burden or post-mortem IL-6 and VEGF concentration. 

We used the syngeneic 4T1 model of human disease which allows for the use of immunocompetent animals [24]. Additional advantageous features of the 4T1 model include metastatic spread in a fashion similar to human disease [25] and the ability to perform post-mortem quantification of tumour cells in solid organ tissues [26].

Hepatic metastatic colony counts, a secondary outcome measure, were not significantly altered by the administration of perioperative propofol, methylprednisolone or lidocaine when compared to control. Currently, we are unable to comprehensively explain this finding, particularly in the presence of a reduced pulmonary tumour burden. 

However, we offer the following tentative hypothesis. The murine 4T1 model preferentially metastasises to the liver and lungs [25] and secondary spread is dependent on the duration from inoculation to cull and specimen retrieval. Previous investigation [26] into 4T1 spontaneous metastatic disease detected pulmonary metastases in 100% of animals at 14 to 18 days post-inoculation but no hepatic metastases were detected at the same time-point. At 22 days post-inoculation, hepatic tumours were detected in 60% of animals. In view of the time-dependent nature of spontaneous spread in the 4T1 model, it may be that organ retrieval two weeks after resection of the induced tumour does not allow a sufficient period of time for differences in hepatic tumours between the groups to be detected due to variable penetrance in the incidence of liver metastases between the experimental groups. 

We found that the use of intravenous propofol as a sole agent for the provision of general anaesthesia in a murine population presented difficulties. Without co-administration of opioids, the depth of anaesthesia achieved with propofol alone, in this particular model, is insufficient to ethically and humanely perform surgical procedures. Where the intravenous dose of propofol is increased to avoid concurrent administration of other agents, cardio-respiratory arrest rapidly ensues. Considering the body of work our research is based on [5,6], we wished to avoid introducing opioids into the model as this would introduce a further confounding variable due to their potential for influencing post-operative oncological outcomes. We developed a reliable model where we could administer intravenous propofol in safe manner provided sevoflurane was concurrently administered to ensure an adequate depth of anaesthesia. 

Where administered as a bolus of 5 mg.kg^−1^ perioperatively, propofol significantly decreased the number of pulmonary metastatic colony counts when compared to a control group. When examined in the context of previous investigation, this result is consistent with the potential anti-tumour effects exhibited by propofol in regards to both immunity [8,27,28] and inflammation [29,30].

A number of mechanisms through which propofol may exert a defence against tumour invasion have been postulated, though it is probable that the beneficial immunological effects of propofol are primarily due to its effect on natural killer (NK) cell function [14]. It may simply be the case that propofol is less harmful than other agents and has been shown to have a lesser impact on NK cell activity in comparison to other commonly used intravenous agents [8]. However, propofol has also been shown to have anti-inflammatory properties [15,17,18] and activity against hypoxia-inducible factors [19]. Existing in vivo and in vitro studies of propofol, using biochemical and clinical endpoints, have unfortunately shown inconsistent results [14,16,17,29,30] and it is difficult to take a definitive position on the anti-tumour effect of propofol. 

It is plausible that propofol could exert an anti-tumour effect at a cellular level even in the absence of a clinically appreciable level of anaesthesia or sedation. Though the clinically obvious sedative and anaesthetic effects from a propofol bolus dose are rapidly terminated, this does not mean that the drug itself has been cleared from the body. It may indicate that blood propofol concentrations are below those necessary to maintain sedation or sleep [31].

Lidocaine is the obvious choice for evaluating amide local anaesthetics [32]. In line with recently published findings [11], we found that the perioperative administration of intravenous lidocaine significantly reduced the pulmonary burden of metastatic disease in an animal model of breast cancer. This finding was consistent with evidence supporting the anti-metastatic effects of amide local anaesthetic agents [11,33]. We initially aimed to continue lidocaine infusions into the post-operative recovery period but this was not feasible, primarily due to logistical concerns about maintaining an infusion in animals emerging from general anaesthesia. 

We found that the perioperative administration of intravenous methylprednisolone significantly increased the metastatic tumour burden of the lungs. No significant difference was found between the control group and the steroid group in terms of hepatic spread. 

It has been previously postulated that neuroendocrine stress responses to surgery temper immune function in the perioperative phase, thereby facilitating the dispersion and metastasis of tumour cells dislodged during surgical resection [34]. This rationale formed the basis for investigation of the potential for beta-blockade and cyclo-oxygenase (COX) inhibition to attenuate the metastatic burden in-vivo, but there has been a no consideration of the role of perioperative steroids in this context of post-operative metastatic disease. 

Prospective randomised clinical trials have shown that pre-operative intravenous methylprednisolone enhances recovery after a variety of non-cancer surgical procedures, with associated reduction in surgical stress response [21,22]. A meta-analysis examining the effect of methylprednisolone in cancer surgery indicated that it reduces stress response cytokine levels and all major morbidities [23]. Conversely, and more specific to malignant disease and spread, perioperative steroid administration modulates the host immune response [9] and suppresses natural killer cell activity, cells which play a pivotal role in immunosurveillance [35]. 

Previous analysis of patients undergoing elective surgery for colon cancer found no differences in 5-year survival between those who received perioperative dexamethasone and those who did not [36] but, there was a significant increase in disease recurrence, with a trend towards increased cancer-specific mortality where dexamethasone was given in comparison to those patients receiving placebo [37].

Cytokine analysis, using ELISA, was carried out on post-mortem serum in order to determine serum interleukin-6 and vascular growth endothelial factor concentration. The blood volume of the animals involved in the model is in the order of 1–2 mL. Sampling blood for serum analysis during anaesthesia would have been a major hypovolaemia stress for the animals and would have risked their early demise. No differences were found across the different groups in relation to IL-6 and VEGF expression. Tumour cell invasion and spread is associated with changes in the cellular and cytokine milieu which involves immense interaction and complexity and is not inherently dependent on any one cytokine. 

## 4. Methodology

A 4T1 murine tumour model was used to represent human breast cancer. Eight- to ten- week old female immunocompetent BALB/c mice were used (Charles River laboratories, Edinburgh, Scotland). The animals were housed within the barrier unit in the Biomedical Facility (BMF), University College Dublin (UCD), in groups of five or less, in an air-conditioned room, at ambient temperatures of 21–22 °C and 50% humidity and under a 12 h light-dark cycle. All animals had ad libitum access to food and water. Upon arrival to UCD BMF, the mice underwent acclimatisation for five days prior to being enrolled into a treatment group. We adhered to the Animal Research: Reporting of In Vivo Experiments (ARRIVE) guidance [38] and our work was conducted in line with EU directive 2010/63. The study protocols were authorised by the Health Products Regulatory Authority of Ireland prior to commencing this research; project authorisation number AE18982/P126, granted on 17th November 2017.

### 4.1. Group Enrolment

Following acclimatisation, mice were randomised into one of four groups. A blocked study design was employed in order to distribute enrolled animals across the duration of the study and throughout the different experimental groups. Throughout the four experimental groups, a total of 72 mice were inoculated, with *n* = 60 available for final analysis after the processing and plating of solid organ tissues. Six animals did not develop tumours amenable to resection, three were found to have extensive intraperitoneal disease at the time of primary excision and one scored excessively high on post-operative checks. These ten animals were humanely euthanised and were not included in any analysis. Data for two animals were unavailable due to inadvertent damage to post-mortem specimen plates which meant they were unavailable for analysis. Table 1.

The treatment groups were as follows:

(a) Control group. Sevoflurane 3% in 50% oxygen-air mixture.

(b) Lidocaine group. Sevoflurane 3% in 50% oxygen-air and intravenous lidocaine; bolus of 1.5 mg.kg^−1^ followed by infusion of 2 mg.kg^−1^.h^−1^.

(c) Propofol group. Sevoflurane 3% in 50% oxygen-air and intravenous propofol; bolus of 5 mg.kg^−1^ over 30 to 60 s.

(d) Methylprednisolone group. Sevoflurane 3% in 50% oxygen-air and intravenous methylprednisolone; bolus of 30 mg.kg^−1^ over 30 to 60 s. 

Where lidocaine was administered, the infusion was continued until cessation of anaesthesia delivery with sevoflurane. The period from induction to cessation of anaesthesia was standardised at 30 min for all animals.

### 4.2. Conduct of Study

See Figure 3.

### 4.3. Study Day 0

The 4T1 cells were cultured in Roswell Park Memorial institute (RPMI) culture medium, with prophylactic penicillin/streptomycin (1%) antibiotic and foetal bovine serum (10%) added. Cells were stored in sterile culture cell flasks at 37 °C and 5% CO_2_. On the day of inoculation, 4T1 cells were removed from the flasks, centrifuged, counted and then re-suspended in RPMI medium free of foetal bovine serum. A final concentration of 1 × 10^6^ cells per millilitre was obtained (Countess Automated Cell Counter, Invitrogen, Carlsbad, CA, USA) and cells put on ice prior to inoculation.

The lower right inguinal mammary gland of the relevant animal was inoculated with 2.5 × 10^4^ 4T1 tumour cells using a 26 G hypodermic needle. Tumour growth was then monitored on a routine basis for one week. Once the tumour became palpable, the diameter of the lesion was measured in two axes using an electronic calliper. The “tumour diameter” was then taken to be the square root of the product of these two dimensions. Animal welfare was monitored using the mouse grimace scale (MGS) [39] and regularly assessing weight, appearance, behaviour and respiratory pattern. 

### 4.4. Study Day 7

Primary excision was performed seven days after initial tumour cell inoculation. Based on previous work within our unit, this timing has the advantage of reducing the risk of spontaneous metastasis while still allowing for a primary tumour of sufficient size at time of surgical resection [11]. The tumour was excised under general anaesthesia with sevoflurane in oxygen-air. Sevoflurane was the sole agent administered for the purposes of anaesthesia induction and maintenance and was across all experimental groups. Sevoflurane (AbbVie, Chicago, IL, USA) was initially delivered into an induction box containing the mouse at a concentration of 5% in a 50% oxygen-air mixture. Once the mouse was determined to have lost the pedal withdrawal response to nociceptive stimulus, the concentration of sevoflurane was reduced to 3%. The delivery of the anaesthetic agent was also changed from induction box to a purpose-built “face-mask” for maintenance during tumour resection. 

The surgical site was prepared with chlorhexidine gluconate and isopropyl alcohol (ChloraPrep^®^, CareFusion, Basingstoke, England) after shaving. Where required, intravenous access was established with a 26 G cannula sited in a lateral tail vein which allowed for continuous infusion of administration of lidocaine. Where a bolus of agent was required, a 26 G needle was used to access the vein and the drug administered over an appropriate duration. Where relevant, intravenous agents were administered in the doses described above. All boluses were administered and infusions commenced prior to any surgical incision. 

Tumour excision was performed using a standard surgical aseptic technique and the surgical site was closed using interrupted 7.0 polypropylene sutures (Ethicon, Somerville, NJ, USA). The duration of the procedure was standardised to 30 min from induction to cessation of anaesthesia. The pedal response to nociceptive stimulus was assessed for a second time prior to commencing surgical excision. Analgesia was administered to all animals subcutaneously prior to commencing tumour resection; paracetamol 200 mg.kg^−1^ and carprofen 10 mg.kg^−1^.

### 4.5. Study Day 7 to Study Day 21

For a minimum of 48 h post-operatively, all animals undergoing surgery were assessed and received analgesia at 12-hourly intervals. Analgesia was standardised to subcutaneous administration of paracetamol 200 mg.kg^−1^ and carprofen 10 mg.kg^−1^. After the first two post-operative days, post-surgical animals were monitored on a daily basis until study day 21 when they were culled. Animal welfare was monitored by assessing weight, appearance, behaviour, respiration and use of the mouse grimace scale (MGS) [39]. Supplemental administration of analgesia was given based on this monitoring. A small number of animals were given supplemental analgesia after the initial 48 h post-operative period. We found no differences between the groups with regards to pain scores, as assessed by MGS, measured on the operative day and first 3 post-operative days. One of the animals in Propofol group did score excessively high in the immediate post-operative period and was euthanised. This animal was excluded from further analysis.

### 4.6. Study Day 21 to Study Day 35

On study day 21, animals were culled by cervical dislocation (without anaesthesia) and then decapitated. The liver, lungs and blood samples were then taken in an aseptic fashion for analysis. The primary endpoint of this study was the number of metastatic colony counts found in lung tissue post-mortem. The lungs were then treated with collagenase type IV (125 units.mL^−1^, Sigma-Aldrich, MO, USA) in Hank’s Balanced Salt Solution (HBSS). The livers were treated with hyaluronidsase (500 units.mL^−1^, Sigma-Aldrich, MO, USA) and collagenase type I (125 units.mL^−1^, Sigma-Aldrich, MO, USA) in HBSS. Both solid organ samples were then passed through a cell strainer in order to better isolate cells. These cells were then cultured for 14 days at 37 °C in air with 5% CO_2_. 60 mM of 6-thioguanine (Sigma-Aldrich, MO, USA) was added to the culture medium, Iscove′s Modified Dulbecco′s Medium (IMDM). 

On study day 35, the cells were fixed using 100% methanol and stained using 0.03% *w/v* methylene blue for five minutes. The culture plates were then subsequently examined by two researchers blinded to the experimental treatment groups. A low magnification microscope was available to count the number of colonies per plate. The mean of the values counted by each researcher was then used for analysis. 

### 4.7. Cytokine Analysis

After culling and decapitation, blood samples were taken in the immediate posthumous period on study day 21, two weeks after resection of the primary tumour. Blood samples were then centrifuged in order to isolate serum. Within two hours of collection, the isolated serum was stored −20 °C until required. Enzyme-linked immunosorbent assay (ELISA) was then employed to detect and quantify interleukin-6 (IL-6) (Abcam, Cambridge, UK) and vascular endothelial growth factor (VEGF) (Sigma-Aldrich, MO, USA). The sera of three individual animals known to have metastatic disease from each group was used. Sera was not mixed to achieve the results. Rather, the cytokine values were calculated for each of the three individual mice. From these samples, 20 μL of serum was diluted to a final volume of 100 μL with the appropriate assay dilutant. After preparation, the samples were then analysed in order to detect and quantify the presence of IL-6 and VEGF. The preparation of ELISA samples and standards was undertaken by a researcher blind to group randomisation. Sample cytokine concentrations were calculated from a best-fit polynomial curve, interpolated from the known concentration of standard solutions against measured absorbance at 450 nm. 

### 4.8. Statistical Analysis

The syngeneic 4T1 murine model has been successfully used previously to examine the influence of perioperative intervention on post-operative tumour burden [11]. This project was designed to detect a reduction in pulmonary metastatic burden of 50% between the experimental groups, operating under the assumption that a halving of the metastatic burden should lead to improved clinical outcome. To achieve 90% power to detect this level of difference and a type I error of 0.05 we calculated that *n* = 15 animals were required per group. This supposes an observed count of 50 pulmonary metastatic colonies in the control group and a standard deviation of 30 colonies. To account for unexpected loss prior to analysis, *n* ≥ 17 animals were inoculated in each group. 

One-way ANOVA analysis was used to compare group weights and tumour sizes at the time of resection on study day 7. The metastatic colonies counted in the solid organ samples were found to not be normally distributed after employment of the d’Agnostino and Pearson omnibus normality test. Therefore, the Kruskal-Wallis test was applied to analyse the differences in metastatic burden between the groups. A one-way ANOVA was employed with analysis of variance and post hoc Bonferroni correction for multiple testing was employed to compare the normally distributed cytokine data.

## 5. Conclusions

In conclusion, using a 4T1 model of breast cancer surgery, this data supports the hypothesis that perioperative interventions influence post-operative outcome. Lidocaine and propofol reduce metastasis, whereas high dose steroid administration increases metastasis. A prospective, randomised clinical trial examining the effect of lidocaine and propofol anaesthetic techniques on long-term oncological outcomes seems warranted. 

## Figures and Tables

**Figure 3:**
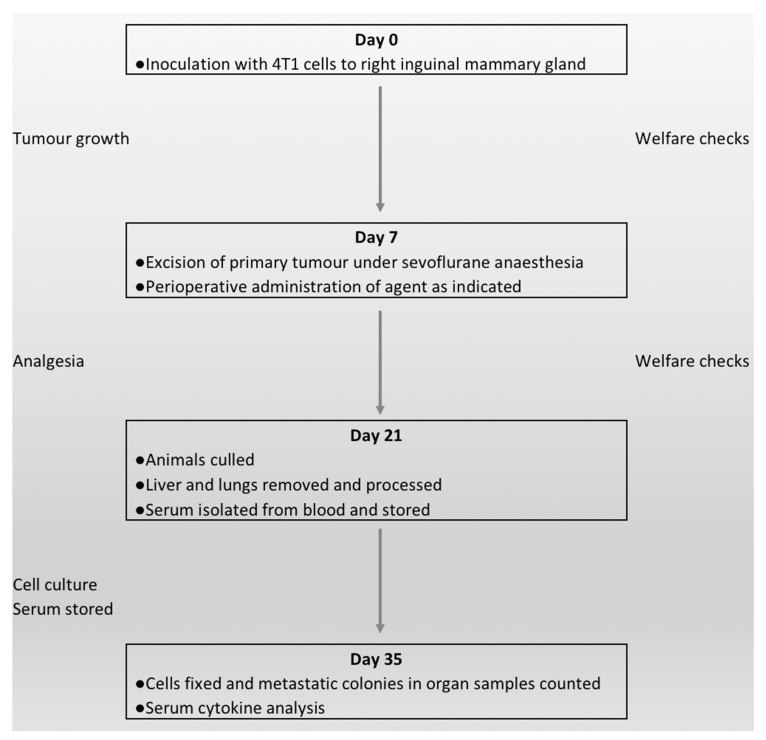
Flow diagram of the work carried out. Study day 0; inoculation of animals, with welfare and tumour checks on a routine basis for one week. Study day 7; excision of the primary tumour. Animals were randomised into one of four groups. Lidocaine, propofol and methylprednisolone was given as indicated. Welfare checks were carried out on a daily basis (as a minimum) until the animals were culled. Analgesia was given routinely in the initial two days post-operatively and then on an “as needed” basis. Study day 21; animals were humanely culled. The entire liver and lungs were taken and then processed and plated for culture. Post mortem blood samples were taken and serum isolated and stored. Study day 35; the cultured solid organ cells were fixed, stained and counted. Analysis of serum cytokines using ELISA.

**Table 1:** Animals employed in study and available for analysis.

	C	L	P	M	Total
Mice Inoculated	17	18	20	17	72
−No tumor	1	2	2	1	6
−I.P tumor	1	1		1	3
−Excessive Score			1		1
−Damaged plates			2		2
Animals Available	15	15	15	15	**60**

72 animals were inoculated. In total, six animals did not develop any tumours. Three mice developed excessively large intraperitoneal (I.P) tumours and were culled under anaesthesia. One animal scored excessively high on post-operative welfare checks and so was humanely euthanised. Plates growing post mortem solid organ colony counts for two animals were inadvertently damaged and so were not available for analysis. 60 animals were therefore available for analysis. C = control group. P = propofol group. L = lidocaine group. M = methylprednisolone group.
